# Rechallenging Fluoropyrimidine-Induced Cardiotoxicity and Neurotoxicity: A Report of Two Cases

**DOI:** 10.7759/cureus.26824

**Published:** 2022-07-13

**Authors:** Sethi Ashish, Moses S Raj, Dulabh Monga, Gene Finley

**Affiliations:** 1 Department of Hematology and Oncology, Allegheny Health Network, Pittsburgh, USA

**Keywords:** infusional 5-fu, bolus 5-fu, cerebellar infarct, capecitabine, cardiotoxicity, 5-fu

## Abstract

Fluoropyrimidines (FP’s) such as fluorouracil (5-FU) and capecitabine are antimetabolites widely used in many solid tumors. FPs side effects are caused mainly by a lack of dihydropyrimidine dehydrogenase (DPD) enzyme. It has been noticed that treatment with infusional regimens of 5-FU is associated with more adverse events (AE) compared to bolus forms. Here, we report two cases of unusual side effects seen with infusional 5-FU and capecitabine and how early intervention by withholding ongoing treatment can help in preventing progression and mortality.

## Introduction

Fluoropyrimidines (FPs) such as fluorouracil (5-FU) and capecitabine are antimetabolites that are widely used in solid tumors and concurrently with external beam radiation as a palliative care management. They are metabolized with dihydropyrimidine dehydrogenase (DPD). The genetic polymorphism in the genes encoding DPD may result in a decrease or loss of enzyme activity which can lead to the accumulation of the chemotherapy and its metabolites causing potential toxicity [1]. The common side effects associated with FPs mainly include nausea, emesis, diarrhea, myelosuppression, and hand-foot syndrome (HFS) [2,3]. FP-induced cardiotoxicity and neurotoxicity are rare but could potentially be detrimental if not recognized early. Here, we report two cases of unusual side effects in the form of cardiotoxicity and acute cerebellar syndrome with FPs and how early intervention helped in preventing progression and mortality.

## Case presentation

Case 1

A 68-year-old female patient with stage IIA anal squamous cell carcinoma (SCC) was on chemotherapy with mitomycin and capecitabine. She had a history of hypertension (HTN) and hyperlipidemia but no prior history of acute coronary syndrome (ACS) or any ischemic event. Also, there was no family history of premature coronary artery disease (CAD) or sudden cardiac death (SCD). Patient never smoked cigarettes or consumed alcohol and recreational drugs.

About four days after the first dose of capecitabine for anal carcinoma, patient experienced severe chest pain which was described as a sharp burning retrosternal pain with no radiation. The pain lasted about 45 minutes continuously and resolved spontaneously and was not associated with shortness of breath, palpitations, or dizziness. Later, a similar pain that lasted 15 minutes prompted her to present to the emergency room (ER). High sensitivity troponin was noted to be elevated at 43 nanograms per milliliter (ng/mL) which subsequently down trended to 21 ng/mL. However, she had no significant electrocardiographic (ECG) changes consistent with ongoing ischemia. Over the course of one week, with multidisciplinary team discussion, there was concern that patient may have been experiencing chemotherapy-induced coronary vasospasm for which she was initiated on isosorbide dinitrate 60 mg prophylactically. Her capecitabine dose of 1500 mg twice a day was changed to 1000 milligrams (mg) in the morning and 2000 mg at night, and then after no episodes of coronary vasospasms were reported. Later isosorbide dinitrate was discontinued.

Case 2

A 54-year-old male patient with stage IIIB, T3 N1 microsatellite adenocarcinoma of distal transverse colon was status pose hemicolectomy. Post-surgery he received four cycles of adjuvant chemotherapy with folinic acid, fluoro­uracil, and oxali­platin (FOLFOX). In infusional 5-FU in cycle 4, the dose was reduced to 20% because of diarrhea. In spite of dose reduction, patient suffered a syncopal episode after which he was admitted to the hospital for further evaluation. The patient reported that his syncopal attack was associated with nausea and vomiting but could not describe whether or not he hit his head. Brain magnetic resonance imaging (MRI) revealed increased signal in the supra-tentorial white matter but no area of restricted diffusion. Diffusion-weighted imaging in posterior fossa including the right cerebellum and cerebellar tonsil was concerning for an acute infarct. Computed tomography (CT) head and CT angiogram of head and neck were unrevealing. Also, transesophageal echocardiogram (TE) and transthoracic echocardiogram (TTE) were negative for cardiac thrombus or patent foramen ovale (PFO). Patient's case was discussed in multidisciplinary rectal cancer tumor board and consensus was to change therapy to a bolus 5-FU strategy since the infusional 5-FU appeared to be causing the thromboembolic event or acute cerebrovascular accident (CVA). Chemotherapy was changed to bolus 5-FU with oxaliplatin and antiplatelet therapy was started in view of cerebellar stroke. Patient remained stable thereafter at the time of reporting this article.

## Discussion

Angina is the most common cardiac manifestation associated with FPs (Table 1). Cardiotoxicity associated with 5-FU is variable and depends mainly on its route of administration. The risk is higher with infusion from 2% to 18%, compared to bolus regimen of 1.6-3% [4,5]. Chest pain due to FPs may be non-specific or associated with ECG changes with or without rise of serum markers of cardiac injury such as troponins and creatine kinase (CK). However, it has also been noticed that the combination of FPs with bevacizumab results in higher incidences of cardiotoxicity [6,7].

**Table 1:** Frequent cardiac complications related to 5-FU administration. The table is adapted from Saif et al. (2009) [8]. VT: ventricular tachycardia; VF: ventricular fibrillation; CHF: congestive heart failure

Cardiac event	Percentage (%)
Angina	45
Myocardial infarction	22
Arrhythmias (atrial fibrillation, VT, and VF)	23
Acute pulmonary edema	5
Cardiac arrest	1.4
Pericarditis	1.4
CHF	2

There are no standard treatment guidelines for managing FP cardiotoxicity. The primary management of 5-FU cardiotoxicity involves stopping the 5-FU infusion and treating the symptoms with antianginal agents such as nitrates and non-dihydropyridine calcium channel blockers (CCBs). Kasi and Gaude safely rechallenged three patients with three-drug cardio-protective regimen which included oral ranolazine 1000 mg BID and amlodipine 2.5 mg [9]. Cardioprotective regimen was given the day before starting 5-FU infusion/oral capecitabine and continued until completion of infusion/treatment [9]. Rechallenge should be performed in a vigilantly monitored setting if no alternatives are available [10]. Dose reduction of FPs, transitioning to a bolus form 5-FU, and prophylactically treating with nitrates or CCBs are the key components while rechallenging patients with FPs after the initial cardiac event [11].

Similarly, FP-induced neurotoxicity due to DPD enzyme deficiency is also rare toxicity. HFS and peripheral neuropathy are the most common neurotoxicity associated with FPs. However, several other neurological side effects such as optic neuropathy, focal dystonia, seizures, and parkinsonian syndrome have been noticed with the use of FPs. Neurologic symptoms occur within three to seven days after starting capecitabine, unlike the neurotoxicity due to FU, which occurs later [12].

High serum ammonia levels due to 5-FU administration have been noticed to be 5.7% (16/280) among cancer patients treated with a 24-hour infusion of 5-FU (2600 mg/m^2^/week) and leucovorin (300 mg/m^2^/week) [13]. Although the actual mechanism of hyperammonemia is unknown, many factors, such as renal dysfunction, constipation, weight loss, and infection, are known to aggravate the condition [14]. Yeh and Cheng proposed two mechanisms for the pathogenesis of hyperammonemic leukoencephalopathy - DPD deficiency and the influence of 5-FU catabolites [15]. Niemann et al. reported a patient who was rechallenged with capecitabine one year after first capecitabine-based therapy [16]. Within six days of the rechallenge, epilepsy-like manifestations consisting of repeated pain and spasms of throat and mandibular muscles appeared. MRI brain revealed diffuse subcortical white matter alterations [16]. Capecitabine was stopped and symptoms resolved within two days and MRI showed complete regression of pathological findings one month later [16]. Supportive care and withholding ongoing palliative treatment with 5-FU due to neurotoxicity is a key approach in preventing progression of acute CVA.

## Conclusions

Rechallenging patients with FPs after developing cardiac and neurotoxicity is a great concern and carries high risk of morbidity and mortality. Switching to a different class of chemotherapy would be an ideal approach in such scenario. However, more research is needed to define the ideal course of treatment guidelines with FP-induced cardiac and neurotoxicity. Also, testing for DPD enzyme deficiency before starting treatment with FPs should be a regular protocol to prevent the possibility of rare side effects which can be easily overlooked by oncologists.
